# Comparison of 1.1 GBq and 2.2 GBq Activities in Patients with Low-Risk Differentiated Thyroid Cancer Requiring Postoperative ^131^I Administration: A Real Life Study

**DOI:** 10.3390/cancers15092416

**Published:** 2023-04-22

**Authors:** Alfredo Campennì, Rosaria Maddalena Ruggeri, Maria Luisa Garo, Massimiliano Siracusa, Giovanna Restuccia, Andrea Rappazzo, Helena Rosarno, Antonio Nicocia, Davide Cardile, Petra Petranović Ovčariček, Sergio Baldari, Luca Giovanella

**Affiliations:** 1 Department of Biomedical and Dental Sciences and Morpho-Functional Imaging, Unit of Nuclear Medicine, University of Messina, 98125 Messina, Italy; 2 Department of Human Pathology DETEV, Unit of Endocrinology, University of Messina, 98122 Messina, Italy; 3 Department of Cardiovascular Research, University Campus Biomedico, 00128 Roma, Italy; 4 Department of Oncology and Nuclear Medicine, University Hospital Center Sestre Milosrdnice, 10000 Zagreb, Croatia; 5 School of Medicine, University of Zagreb, 10000 Zagreb, Croatia; 6 Clinic for Nuclear Medicine and Competence Centre for Thyroid Diseases, Imaging Institute of Southern Switzerland, Ente Ospedaliero Cantonale, 6500 Bellinzona, Switzerland; 7 Clinic for Nuclear Medicine, University Hospital and University of Zurich, 8006 Zurich, Switzerland

**Keywords:** radioiodine therapy, low-risk DTC, radioiodine ablation, low radioiodine activity, moderate radioiodine activity, differentiated thyroid carcinoma

## Abstract

**Simple Summary:**

The aim of the present study was to retrospectively evaluate the efficacy of low (1.1 GBq) versus moderate (2.2 GBq) ^131^I activities in a large series (n = 299) of low-risk differentiated thyroid carcinoma (DTC) patients requiring postoperative ^131^I ablation. At the follow-up, according to the ATA criteria, an excellent response was observed in 96.9% of patients treated with moderate ^131^I activities versus 85.6% of patients treated with low ^131^I activities (*p* = 0.029). Conversely, a biochemically indeterminate or incomplete response was observed in 22.2% of patients treated with low ^131^I activities versus 1.8% of patients treated with moderate ^131^I activities (*p* = 0.001), and an incomplete structural response was observed in three patients treated with low ^131^I activities versus two patients treated with moderate ^131^I activities (*p* = 0.654). In conclusion, we encourage the use of moderate instead of low activities when ^131^I ablation is indicated in order to reach an excellent response in a significantly larger proportion of patients, including patients with the unexpected persistence of the disease.

**Abstract:**

Objectives: To compare the efficacy of low and moderate ^131^I activities in low-risk differentiated thyroid carcinoma (DTC) patients requiring postoperative thyroid remnant ablation in a real-world clinical setting. Methods: We retrospectively reviewed the records of 299 low-risk DTC patients (pT1-T2, Nx(0) Mx) who had undergone (near)-total thyroidectomy followed by ^131^I therapy, using either low (1.1 GBq) or moderate (2.2 GBq) radioiodine activities. The response to initial treatments was evaluated after 8–12 months, and patient responses were classified according to the 2015 American Thyroid Association guidelines. Results: An excellent response was observed in 274/299 (91.6%) patients, specifically, in 119/139 (85.6%) and 155/160 (96.9%) patients treated with low and moderate ^131^I activities, respectively (*p* = 0.029). A biochemically indeterminate or incomplete response was observed in seventeen (22.2%) patients treated with low ^131^I activities and three (1.8%) patients treated with moderate ^131^I activities (*p* = 0.001). Finally, five patients showed an incomplete structural response, among which three and two received low and moderate ^131^I activities, respectively (*p* = 0.654). Conclusions: When ^131^I ablation is indicated, we encourage the use of moderate instead of low activities, in order to reach an excellent response in a significantly larger proportion of patients, including patients with the unexpected persistence of the disease.

## 1. Introduction

Thyroid cancer is the most common endocrine malignancy, accounting for about 1% of all cancers [1]. Differentiated thyroid cancer (DTC) represents the most common form (80–85% of all thyroid cancers), and its incidence has increased over time, especially affecting women with small-sized papillary histotype [2,3,4,5,6]. Thyroid surgery followed by risk-adapted postoperative ^131^I therapy and levothyroxine therapy personalized according to the patient’s risk of recurrence is considered the current standard of care, leading to an excellent response in most (>80%) DTC patients [7]. 

However, the 2015 American Thyroid Association (2015 ATA) guidelines recommend de-escalated treatment strategies for many DTC patients [8]. Accordingly, ^131^I therapy was indicated in DTC patients classified to have a high-risk tumor, optionally considered in many low- to intermediate-risk cases, while it was generally discouraged in patients affected by low-risk DTC, with the exception of patients requiring thyroid remnant ablation (i.e., to simplify the follow-up) and those with additional risk factors.

Considering the disputes on such guidelines and consequent differences in clinical practice [9,10], the European Association of Nuclear Medicine (EANM), the Society of Nuclear Medicine and Molecular Imaging (SNMMI), the European (ETA), and the American (ATA) Thyroid Association organized a meeting in 2018 to discuss the use of ^131^I in the DTC management [11]. First, it was recognized that “the actual goal of ^131^I therapy can only be determined once the postoperative disease status has been assessed”, and the evaluation of multiple factors beyond pathology-based risk stratification was recommended. Accordingly, low-risk DTC patients may require adjuvant or even curative ^131^I based on additional risk factors (i.e., patients with additional risk factors or patients requiring maximal treatment) and postoperative assessment (i.e., high postoperative thyroglobulin levels). Similar positions were recently expressed by the European Thyroid Association (ETA) [12], the Society of Nuclear Medicine and Molecular Imaging (SNMMI), and the European Association of Nuclear Medicine (EANM) [13].

Additionally, when ^131^I adjuvant therapy is required in selected low-risk patients [8,11,12,14], the optimal ^131^I activity to be administered remains largely debated. In fact, two large prospective randomized trials [15,16] have demonstrated the non-inferiority of ^131^I 1.1 GBq compared to ^131^I 3.7 GBq for ablation. However, in low-risk patients with additional risk factors, an adjuvant aim should be pursued [1,11]. Indeed, while the administered ^131^I activity of 1.1 GBq for ablation is an accepted standard, the optimal activity for adjuvant treatment cannot be definitely determined from the published literature [11]. Accordingly, different activities are proposed in different guidelines, namely, 1.1 to 5.5 GBq (ATA), 1.85 to 3.7 GBq (SNMMI/EANM), and 1.1 to 3.7 GBq (ETA) as uncertainties remain regarding differences in clinical conditions (e.g., surgeon volume, quality of preoperative and postoperative assessment) [17,18,19].

Therefore, the present study aims to compare the efficacy of ^131^I 1.1 GBq and 2.2 GBq in low-risk DTC patients requiring postoperative ^131^I adjuvant therapy in a large series of patients managed in a real-world clinical setting. 

## 2. Patients and Methods 

### 2.1. Study Design, Subjects, and Data Collection

Electronic medical records of adult DTC patients (≥18 years) referred to the Nuclear Medicine Unit of the “G. Martino” University Hospital of Messina (Italy) were reviewed. Consequently, patients with low-risk DTC (pT1-T2, Nx (0), Mx) who had undergone ^131^I therapy from 2016 to 2020 were included. On the contrary, low-risk patients treated without ^131^I therapy, all patients with intermediate risk, high risk and poorly DTC, and patients with positive anti-thyroglobulin antibodies (TgAb) were excluded. This study was conducted in accordance with the Declaration of Helsinki. In addition, the protocol was approved by the Ethics Committee of the “G. Martino” University Hospital (Project identification code 1917; 17 March 2017). All subjects gave their informed consent for their inclusion prior to enrollment in this study. During the study period, a total of 669 patients with thyroid disease underwent surgical therapy; of these, 299 DTC patients met the inclusion criteria.

### 2.2. Patient Management, Response Assessment, and Follow-Up

All patients underwent (near)-total thyroidectomy ((n)-TT) and started levothyroxine (LT4) therapy (1.5–1.8 mg/kg body weight). Serum TSH, Tg, and TgAb measurements and neck ultrasound (nUS) were performed 1 to 3 months after surgery. Thereafter, all cases were discussed in our local thyroid tumor board meetings, and patients were managed according to multidisciplinary advice. Patients selected for ^131^I ablation underwent recombinant human TSH (rhTSH) stimulation according to standard protocol (i.e., intramuscular injection of 0.9 mg rhTSH daily for two consecutive days) [20,21] and received low (i.e., 1.1 GBq) or moderate (i.e., 2.2 GBq) ^131^I activity at the discretion of the attending physician. A post-therapy whole-body scan (PT-WBS), coupled with single photon emission tomography-computed tomography (SPECT/CT) (i.e., pT-imaging), was performed in all patients 2 to 5 days after ^131^I therapy, as previously reported [2,22,23]. In all patients, the first follow-up was obtained three months after ^131^I therapy by clinical evaluation and basal laboratory tests (i.e., TSH, free triiodothyronine (FT3), free thyroxine (FT4), Tg, and TgAb). The response to initial treatments was assessed 8 to 12 months after ^131^I therapy by laboratory testing (both basal and after rhTSH administration), nUS, and ^123^I diagnostic whole-body scan (^123^I-Dx-WBS) with SPECT/CT (i.e., ^123^I-Dx-imaging) as described below.

### 2.3. Laboratory Tests

Serum Tg and TgAb values were measured on day 1 (basal), day 3 (early stimulated Tg), and day 5 (late stimulated Tg) during rhTSH stimulation. Serum TSH and TgAb were measured at the Core Laboratory of the “G. Martino” University Hospital of Messina (Italy) by fully automated Access^®^ TSH immunochemiluminescent assay (Beckman Coulter, US; functional sensitivity 0.01 mUI/L, reference range 0.4–4.2 mIU/L) and Elecsys^®^ TgAb chemiluminescence immunoassay (Roche Diagnostics, Switzerland; functional sensitivity 40 IU/mL, reference range < 115 IU/mL), respectively. For the purposes of the present study, a sample of each patient’s serum was frozen and sent out to the Department of Laboratory Medicine of the Ente Ospedaliero Cantonale (EOC) of Bellinzona (Switzerland) to measure serum Tg on the Kryptor^®^ Compact Plus platform (BRAHMS Thermo Fisher Scientific, Hennigsdorf, Germany). The Kryptor^®^ hTg sensitive assay is calibrated against the BCR^®^ 457 international reference standard, and its declared functional sensitivity is 0.15 µg/L (Instructions for Use, ThermoFisher, Waltham, MA, USA). To conform to 2015 ATA response criteria, Tg values below 0.2 were transformed into 0.20 ng/mL for statistical analysis purposes.

### 2.4. Imaging

Neck ultrasound (nUS) was carried out by expert ultrasonographers using a high-resolution ultrasound system (Logiq3 Expert, GE Healthcare, Little Chalfont, UK or ACUSON 3000 Siemens, Erlangen, Germany) equipped with high energy linear probes (14 MHz);

The ^123^I-Dx-WBS with SPECT/CT (i.e., ^123^I-Dx-imaging) was obtained using a double-headed gamma camera (Xeleris, GE Medical System, Chicago, IL, USA) equipped with high-energy low-resolution parallel hole collimators (HELRPAR) and was performed as previously reported [2,24].

In particular, whole-body images were obtained from head to proximal thighs (anterior and posterior views, matrix 256 × 256, magnification 1, acquisition time 10 cm/min). Static images of the neck and thorax (anterior and posterior views, magnification 1, matrix 256 × 256, frame time 900 s) were also acquired. SPECT/CT imaging (i.e., hybrid imaging) was obtained in step-and-shoot acquisition mode (40 s per step, 6° angle, 30 steps per detector) while CT images were acquired under the following conditions: tube voltage of 120 kV, tube current of 80–210 mA, helical thickness of 2.5 mm, table speed of 37 mm/s, table feed per rotation of 18.75 mm/rot, tube rotation time of 0.8 s, and pitch of 0.938:1. All patients were required to drink at least 1.5 L of water and take laxative drugs 1 day before the examination in order to achieve a better target/background ratio.

Finally, patient responses were classified as an excellent response (i.e., stimulated Tg less than 1 ng/mL, and no loco-regional and/or distant metastases at morphological and/or functional imaging), biochemically indeterminate or incomplete response (i.e., negative imaging and stimulated Tg between 1 and 10 ng/mL, or more than 10 ng/mL), and incomplete structural response (i.e., evidence of structural disease regardless of Tg and TgAb results) according to the 2015 ATA guidelines [8].

## 3. Statistical Analysis

All statistical analyses were performed using STATA17 (StataCorp., College Station, TX, USA). Descriptive statistics were reported as median and range (minimum and maximum) for quantitative data, whereas relative frequencies and percentages were used for qualitative data. Patients’ age was also reported as mean and standard deviation given the normal distribution of the variable. For all quantitative variables, the normal or non-normal distribution was verified by the Shapiro–Wilk test. Comparisons between group A (patients with metastatic disease who underwent ^131^I therapy with low therapeutic ^131^I activity) and group B (patients with metastatic disease who underwent ^131^I therapy with moderate therapeutic ^131^I activity) were performed using the Mann–Whitney U test in the presence of quantitative variables, while Fisher’s exact test or likelihood ratio chi-square were performed in the case of qualitative variables. Stepwise-ordered logistic regression was performed to assess the probability of an excellent response as a function of the two different ^131^I activities (1110 MBq versus 2220 MBq). For this purpose, the dependent variable—response to initial treatment—was ordered in the following manner: incomplete structural response = 1, biochemically indeterminate or incomplete response = 2, excellent response = 3. Gender, age, histology, and histotype were included in the model as covariates and subsequently excluded. The significance level for variable removal was set at 5% (*p* < 0.05). The parallel regression assumption for the ordered logistic regression was verified. Statistical significance for comparisons was set at 5% (*p* < 0.05). A power post hoc analysis was performed using logistic regression results.

## 4. Results

Six hundred and sixty-nine DTC patients were managed in our institution from 1 March 2017, through 30 June 2021. Two hundred and ninety-nine patients with low-risk DTC treated with ^131^I therapy were enrolled in this study. Seventy-four TgAb-positive patients, seventy-one low-risk patients treated with surgery alone, one hundred and eighty-eight intermediate-risk patients and thirty-seven high-risk DTC patients were excluded from the present study. In the standard practice, our thyroid tumor board suggests ^131^I therapy in patients with aggressive histological variants (e.g., sclerosing variant, tall-cell variant, hobnail variant), cancer diameter more than 20 mm, isthmic cancer location, bilateral cancer, multifocal cancer with a total tumor diameter of more than 10 mm, and patients referred by low-volume surgical centers. Among the enrolled two hundred and ninety-nine low-risk DTC patients, two hundred and forty-six (82%) had a papillary thyroid carcinoma (PTC) (F = 190, M = 56; F:M = 3.3:1; mean age ± SD = 49.5 ± 12.2, median = 50, range = 20–88); forty-seven (16%) had a follicular thyroid carcinoma (FTC) (F = 30, M = 17; F:M = 1.7:1; mean age ± SD = 51.5 ± 13.9, median = 53, range = 25–79) and six (2%) had a Hurthle cell carcinoma (HC) (F = 5, M = 1; F:M = 5:1; mean age ± SD = 47.3 ± 15.9, median = 49, range = 18–69). Finally, aggressive PTC variants were found in 11 out of 246 patients (4.5%). Regarding the treatment, 139 (46.5%) patients received low ^131^I activity (mean = 1.2362 ± 0.123 GBq; median = 1.236 GBq; range = 1.110–1.376), while in the remaining 160 patients (53.5%), moderate ^131^I activity (mean = 2.235.3 ± 0.888 GBq; median = 2.220 GBq; range = 2.220–2.812) was administered. As shown in Table 1, no differences were found in gender, age, cancer size, histotype, histological aggressive features, and postoperative Tg values.

At the time of pT-imaging, 246 (82.3%) patients (F = 185, M = 61; F:M = 3.0:1; mean age ± SD = 49.4 ± 12.8, median = 51, range = 18–87) showed thyroid remnants alone, while 53 (17.7%) patients (F = 40, M = 13; F:M = 3.1:1; mean age ± SD = 51.7 ± 11.5, median = 51, range = 26–88) showed thyroid remnants and metastases (i.e., loco-regional lymph-node metastases, n = 51; lung metastases, n = 1; lymph-node and lung metastases, n = 1). Among the 53 patients carrying metastases, 22 (41.5%) received low ^131^I activities, while 31 (58.5%) received moderate ^131^I activities (*p* = not significant).

**Figure 1:**
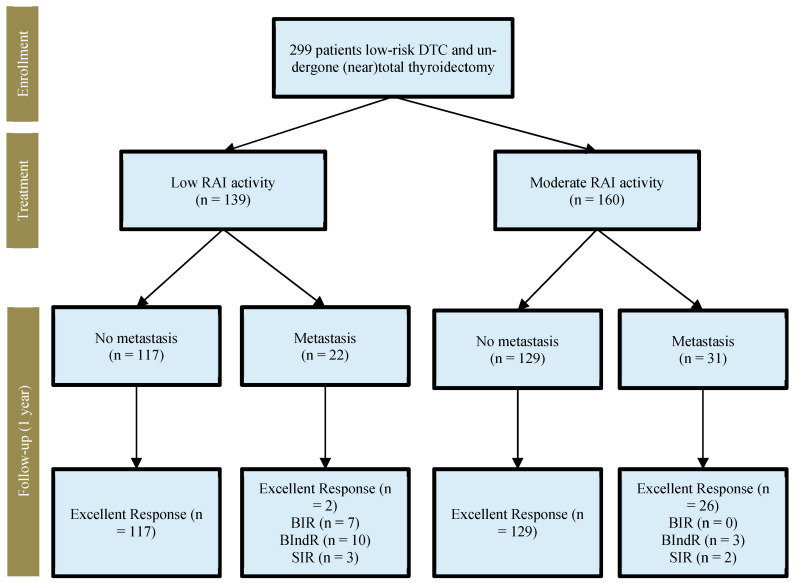
Study flowchart.

At the follow-up (Figure 1), an excellent response was observed in 274 out of 299 (91.6%) patients, specifically, in 119 out of 139 (85.6%) and 155 out of 160 (96.9%) patients treated with low and moderate ^131^I activities, respectively (*p* = 0.029). A biochemically indeterminate or incomplete response was observed in seventeen (22.2%) and three (1.8%) patients treated with low and moderate ^131^I activities, respectively (*p* = 0.001). Finally, five patients showed an incomplete structural response, among which, three and two patients received low and moderate ^131^I activities, respectively (*p* = 0.654). The overall results are summarized in Table 2.

Notably, all patients having thyroid remnants alone reached an excellent response to the initial treatments at the follow-up, independently from the administered activity. On the contrary, twenty-five out of fifty-three (47.2%) patients carrying metastatic disease showed less than excellent response at the follow-up (20 out of 22 (91%) treated with low ^131^I activity and five out of thirty-one (16%) treated with moderate activity, *p* < 0.0001). No differences were found between these patients before treatment (Table 3). 

On the whole, after a stepwise-ordered logistic regression was performed, the final model showed a positive impact of moderate ^131^I activity on excellent response rate in patients with and without metastases. Specifically, the probability to achieve an excellent response in patients who underwent ^131^I therapy using low ^131^I activity was 90%, while the probability to achieve an excellent response in patients who underwent ^131^I therapy using moderate ^131^I activity was 98%. Moreover, basal Tg levels positively affected the excellent-response achievement in the overall patient sample, while no effect was reported in patients with metastases (Table 4). Ninety percent of power emerged from a post hoc logistic regression analysis.

Finally, as per local protocols, patients with incomplete structural response were treated by surgery and cured in all cases (Vice versa, patients with a biochemically indeterminate/incomplete response underwent the second ^131^I therapy with ≥3.7 GBq at the discretion of the attending physician (cumulative activity ≥ 4.81 GBq). Among them, thirteen patients (75.5%) reached an excellent response, while a biochemically indeterminate response persisted in four patients (24.5%). 

## 5. Discussion

Thyroid surgery followed by risk-adapted ^131^I therapy represent the treatment of choice for most DTC patients [13,20]. In the past, ^131^I therapy was routinely performed to destroy thyroid remnant tissue (i.e., thyroid remnant ablation) also in low-risk DTC patients with the aim to simplify the follow-up of such patients by increasing the specificity and accuracy of basal and/or stimulated Tg measurements [17,25]. More recently, the 2015 ATA guidelines underscored the role of ^131^I therapy, recommending its more selective use [8]. In particular, ^131^I therapy was certainly indicated in DTC patients having a high-risk tumor, while it was not indicated or discouraged in low-risk DTC patients (especially without aggressive features and/or vascular invasion) and in most intermediate-risk ones [8]. However, the 2015 ATA guidelines were not endorsed by EANM [10]. In consequence, a large discussion on this topic started among the main involved societies (i.e., ETA, ATA, EANM, and SNMMI) producing a shared document (i.e., the so-called Martinique report) [11] in which the aim of radioiodine therapy was better defined in ablative, adjuvant, or therapeutic treatment. In addition, also in low-risk DTC patients, the choice for or against ^131^I therapy should be always evaluated by an accurate postoperative assessment taking into account several local factors (e.g., quality of pre- and postoperative diagnostic course such as surgeon experience, patients’ preference) other than histopathological report [11]. In this light, a more recent ETA consensus statement [12] suggested a selective use of radioiodine therapy in low-risk DTC, while a greater use of radioiodine therapy was suggested by the SNMMI/EANM guidelines [13]. 

Although disease-specific mortality and recurrence risk are very low in patients with low-risk DTC, ^131^I ablation is recommended in selected cases (i.e., Tg levels greater than 5–10 ng/mL when the likelihood of finding foci of radioiodine uptake outside the thyroid bed is significant) [26]. To date, no consensus has been reached on the optimal activity of ^131^I ablation after near-total thyroidectomy or total thyroidectomy in low-risk DTC patients. While the role of low or high ^131^I activity in intermediate-risk patients has been extensively reported [27] as well as the comparative effectiveness of low or high ^131^I activity in terms of the disease persistence at 10 years in low-risk patients [28], the effect of moderate ^131^I activity is less clear. According to large randomized trials, low ablation activity is as effective (i.e. noninferior radioiodine therapy) as high ablation activity in low-risk DTC patients, with beneficial side effects, reduced radiation exposure, and overall improvement in the quality of life, without discounting the economic benefits in terms of financial costs to the overall health care system [15,16]. However, in the real clinical scenario, the approach to ^131^I activity seems to be more complex because of the presence of a series of confounding factors that could alter the recommended approaches.

Cancer molecular profiling was also proposed to refine DTC risk stratification and ^131^I indications [29]. In particular, BRAF V600E and TERT mutations are frequently reported in a subgroup of cancers with more aggressive clinicopathological behaviors [30]. However, the need for routine genotyping of DTCs has not been definitely established, and such data are not available for our patients [14].

Overall, our patients treated with intermediate ^131^I activities had a significantly higher rate of excellent response compared to the patients treated with low activities. Notably, however, incomplete structural response rates were comparable in both groups. A higher rate of biochemically indeterminate or incomplete response was found in patients treated with low ^131^I activity (22%) compared to those treated with moderate ^131^I activity (1.8%). Notably, thyroglobulin can continue to decrease for a long time after ^131^I therapy, and patients with an incomplete and indeterminate biochemical response can spontaneously reach an excellent response. Accordingly, a long follow-up is necessary before making a definitive conclusion.

However, a biochemically incomplete or indeterminate response may require a more intensive follow-up and, in selected cases, additional treatments, taking into account the associated patients’ distress and financial costs for the healthcare system.

Furthermore, preoperatively unknown metastases (mostly lymph nodes metastases) were detected by PT-WBS in 53 patients (≈18%) of our series. A significantly lower number of lymph node metastases was reported, of course, when prophylactic lymph node dissections were systematically performed [31].

The presence of cervical lymph node metastases does not affect the overall survival, but it may negatively influence the disease-free survival and the quality of life [2,31]. Whether an aggressive surgical approach is justified to avoid the adjuvant administration of ^131^I is debatable, and differences in local factors should be considered before translating the literature into clinical recommendations [32].

Our results are in line with the previous ones, showing a higher tumoricidal effect of moderate activities in low-risk patients with persistent lymph node metastases with a likelihood of an excellent response rate of 98% versus a probability of 90% in patients treated with low ^131^I activity [33,34].

By all means, some limitations of our study should be addressed. First of all, as this was a retrospective study, it was hampered by a series of intrinsic weaknesses, such as selection bias. Secondly, administered activities were decided at the discretion of the attending physician, thus allowing relevant differences between groups of patients. However, no significant differences in demographic and clinicopathological variables were observed in patients treated with low and moderate activities, making major differences unlikely. Thirdly, the power of the present study suffers from a very low number of non-ER events and incomplete structural responses, precluding us from performing a powerful statistical analysis. Conversely, however, any effect already showing statistical significance despite this very low number of events is likely to be not just statistically significant, but also clinically relevant. Nonetheless, considering its discussed limitations, the present results require to be further and, preferably, prospectively confirmed. Finally, the second ^131^I therapy was performed in patients with a biochemically indeterminate or incomplete response at the first follow-up, according to the local protocols. However, the actual need for such procedure in this setting is currently debated, with a growing trend toward a more selective use of the second treatments.

## 6. Conclusions

The incidence of lymph node metastases may be unexpectedly high in low-risk DTC patients in real-life settings. The use of postoperative ^131^I administration should be always considered taking into account local epidemiology, available resources, characteristics and preferences of the patient. When ^131^I ablation is indicated, low and moderate activities are equally effective in terms of structural responses, but moderate activity can quickly reduce thyroglobulin levels and produce an excellent response.

## Figures and Tables

**Table 1:** Demographic, clinical, and pathological characteristics.

	Total Sample (n = 299)		Low RAI Activity (n = 139)		Moderate RAI Activity (n = 160)		*p*-Value
**Gender, n (%)**							
**Male**	74	(24.8)	37	(26.6)	37	(23.1)	0.286 ^§^
**Female**	225	(75.3)	102	(73.4)	123	(76.9)	
**Age (years), median (min–max)**	51	(18–88)	49	(23–79)	52	(18–88)	0.067 *
**Size (mm), median (min–max)**	9	(1–35)	10	(2–35)	8	(1–34)	0.348 *
**Basal Tg (ng/mL), median (min–max)**	0.20	(0.20–3.30)	0.20	(0.20–3.20)	0.20	(0.20–3.30)	0.348 *
**Histology, n (%)**							
**PTC**	246	(82.3)	109	(78.4)	137	(85.6)	0.662 ^ç^
**FTC**	47	(15.7)	25	(18.0)	22	(13.8)	0.084 ^ç^
**HC**	6	(2.0)	5	(3.6)	1	(0.6)	0.088 ^ç^
**Histotype, n (%)**							
**Nonaggressive ^#^**	282	(94.3)	127	(91.4)	155	(96.9)	0.095 ^ç^
**Aggressive ^##^**	17	(5.7)	12	(8.6)	5	(3.1)	0.085 ^ç^
**Metastases pT-imaging, n (%)**							
**No**	246	(82.3)	117	(84.2)	129	(80.6)	0.444 ^ç^
**Yes**	53	(17.7)	22	(15.8)	31	(19.4)	0.215 ^ç^

^131^I RAI—radioactive iodine, PTC—papillary thyroid cancer, FTC—follicular thyroid cancer, HC—Hurthle cell. ^#^ Classical variant, minimally invasive FTC; ^##^ sclerosing variant, tall-cell variant, hobnail variant. * Mann–Whitney U test, ^§^ Fisher’s exact, ^ç^ likelihood ratio chi-square.

**Table 2:** Response to initial treatment according to administrated activities.

	Low ^131^I Activity (n = 139)		Moderate ^131^I Activity (n = 160)		*p*-Value
**ER, n (%)**	119	(85.6)	155	(96.9)	0.029 ^ç^
**BIR, n (%)**	7	(5.0)	0	(0.0)	0.002 ^ç^
**BIndR, n (%)**	10	(7.2)	3	(1.8)	0.046 ^ç^
**SIR, n (%)**	3	(2.2)	2	(1.3)	0.654 ^ç^

ER—excellent response, BIndR—biochemically indeterminate response, BIR—biochemically incomplete response, SIR—structurally incomplete response. ^ç^ Likelihood ratio chi-square.

**Table 3:** Comparison of demographic, clinical, and pathological characteristics of DTC patients with metastases treated with low and moderate ^131^I activities.

	Low RAI Activity (n = 22)		Moderate RAI Activity (n = 31)	*p*-Value	
**Gender, n (%)**					
**Male**	7	(31.8)	6	(19.4)	0.236 ^§^
**Female**	15	(68.2)	25	(80.6)	
**Age (years), median (range)**	49	(32–68)	52	(26–88)	0.539 *
**Size (mm), median (range)**	8	(2–24)	10	(2–21)	0.589 *
**Basal Tg (ng/mL), median (range)**	0.2	(0.2–3.2)	0.3	(0.2–3.3)	0.247 *
**Histology, n (%)**					
**PTC**	21	(95.5)	25	(83.3)	0.226 ^§^
**FTC**	1	(4.5)	5	(16.7)	
**Histotype, n (%)**					
**No aggressive**	20	(90.9)	30	(96.8)	0.563 ^§^
**Aggressive**	2	(9.1)	1	(3.2)	

Tg—thyroglobulin; PTC—papillary thyroid carcinoma; FTC—follicular thyroid carcinoma. * Mann–Whitney U test, ^§^ likelihood ratio chi-square.

**Table 4:** Ordered logistic regression outcome: response to initial treatments in patients with metastatic disease at pT-imaging.

	Overall Sample OR	Patients with Metastases OR
Moderate ^131^I Activity	5.1 *** (2.9)	22.5 *** (17.4)
Basal Tg (ng/mL)	0.1 *** (0.1)	0.2 (0.1)
LR chi-square	53.6 ***	43.12 ***
No. of patients	299	53

*p* < 0.01—Standard errors are reported in parentheses. OR—odds ratio. The ORs were determined through a stepwise ordered logistic regression—a stepwise method for variables removal: gender, age, histology, and histotype were included in the model and subsequently excluded considering a significance level of 0.05 for removal. Covariates included in the initial model gender, age, histology, and histotype. ***: *p* < 0.0001

## Data Availability

The datasets generated during and/or analysed during the current study are available from the corresponding author on reasonable request.
